# Unraveling Caspofungin Resistance in Cryptococcus neoformans

**DOI:** 10.1128/mBio.00156-21

**Published:** 2021-03-16

**Authors:** Nicolas Papon, Gustavo H. Goldman

**Affiliations:** a Host-Pathogen Interaction Study Group (GEIHP, EA 3142), Université Angers, Université Brest, Angers, France; b Federative Structure of Research Cellular Interactions and Therapeutic Applications, SFR 4208 ICAT, Université Angers, Angers, France; c Faculdade de Ciências Farmacêuticas de Ribeirão Preto, Universidade de São Paulo, Ribeirão Preto, Brazil

**Keywords:** *Cryptococcus neoformans*, caspofungin, antifungal resistance, cell wall, posttranscriptional regulation

## Abstract

Cryptococcus neoformans is a basidiomycetous yeast responsible for hundreds of thousands of deaths a year and is particularly threatening in immunocompromised patients. There are few families of antifungals that are available to fight fungal infections, and the unique efficient treatment for the most deadly cerebral forms of cryptococcosis is based on a combination of 5-fluorocytosine and amphotericin B. The toxicities of both compounds are elevated, and more therapeutic options are urgently needed for better management of life-threatening cryptococcosis. The newest class of antifungals, i.e., echinocandins, has initially led to great hope. Unfortunately, C. neoformans was rapidly confirmed to be naturally resistant to these molecules, notably caspofungin. In this respect, we discuss here the recent key findings of the Panepinto research group published in *mBio* (M. C. Kalem et al., mBio 12:e03225-20, 2021, https://doi:10.1128/mBio.03225-20) that provide an unprecedented view of how C. neoformans regulates caspofungin resistance through a complex posttranscriptional regulation of cell wall biosynthesis genes.

## Abstract (precis)

Cryptococcus neoformans is a basidiomycetous yeast responsible for hundreds of thousands of deaths a year and is particularly threatening in immunocompromised patients. There are few families of antifungals that are available to fight fungal infections, and the unique efficient treatment for the most deadly cerebral forms of cryptococcosis is based on a combination of 5-fluorocytosine and amphotericin B.

## COMMENTARY

Like bacterial or viral infections, fungal diseases represent a major problem in human health (1, 2). These yeast and mold infections are particularly worrisome in the most vulnerable subjects, mainly immunocompromised patients, in whom they usually develop as deadly deep-seated infections. Although the epidemiology of these fungal diseases has substantially changed in the past few decades, Candida albicans and Aspergillus fumigatus but also Cryptococcus neoformans remain the prominent causative agents of the most life-threatening forms and are responsible for 1 million deaths a year worldwide (3). In the field of medical mycology, C. neoformans is one of the species that are well documented for standing out biologically from the other pathogenic fungi, which are predominantly Ascomycota, insofar as this yeast is phylogenetically related to the Basidiomycota. This is reflected in its particular ecology, its unique morphology (i.e., a capsulated spherical yeast), its genetics, and in its antifungal susceptibility profile (4–6). Like Pneumocystis jirovecii, C. neoformans contributed to bleak years when AIDS emerged in the 1980s, the latter killing most of the immunocompromised patients with AIDS. Nowadays, although things are getting better since the introduction of the systematic anti-HIV tri-therapy, this opportunist yeast still accounts for more than 600,000 fatal cases of meningitis a year, with marked and increasing impact in transplant patients (2). While C. neoformans is naturally resistant to azole antifungals (e.g., fluconazole, inhibiting ergosterol biosynthesis), the deep-seated forms of cryptococcosis are commonly treated first with a combination of the prodrug 5-fluorocytosine (inhibiting the pyrimidine salvage pathway) and amphotericin B (disrupting ergosterol functions by directly binding this essential sterol) (7). Because of the virulence of this fungal agent and the toxicity of this limited therapeutic option, C. neoformans is still responsible for 15% of AIDS-related deaths (7, 8). Yet, from the beginning of the 2000s, unprecedented hopes arose following the availability of a new class of highly active antifungals, namely, echinocandins. Unfortunately, as soon as the 2010s, it became rapidly well-ingrained in the literature that C. neoformans is naturally resistant to echinocandins (9). However, and intriguingly, preliminary investigations challenging the protein sequence of the echinocandin fungal target, β-1,3-glucan synthase, encoded by the *FKS1* gene, revealed that resistance may not rely on specific amino acid variations, as previously observed in *Candida* and *Aspergillus* species. *In vitro* experiments showed that C. neoformans 1,3-β-glucan synthase activity is very sensitive to caspofungin and cilofungin, suggesting that echinocandin resistance may be due to a mechanism unrelated to echinocandin inhibitors (9). Obviously, this led some research groups to the identification of new candidate mechanisms that may underlie echinocandin resistance in C. neoformans, such as cell wall remodeling and integrity pathways governing, in particular, chitin contents. In such a perspective, identifying molecular components of these regulatory pathways emerged as the main interest since they may represent new targets whose inhibition may potentiate the activities of echinocandins (10).

In this respect, several studies demonstrated a crucial role of calcineurin signaling pathways in the regulation of echinocandin resistance, intracellular trafficking, cell integrity, and RNA processing (11). These observations progressively propelled calcineurin as a pivotal stress-integrating hub regulating a plethora of cell processes, including drug tolerance in connection with cell wall composition. In this context, the research group of John C. Panepinto previously reported the involvement of the pumilio domain and FBF (PUF) domain-containing RNA-binding protein Puf4 in the regulation of endoplasmic reticulum stress in C. neoformans (12). Given that Puf4 has also been shown to act as an effector of calcineurin signaling, this RNA binding protein has cast its spell on the preliminary experiments of Kalem and colleagues.

**FIG 1:**
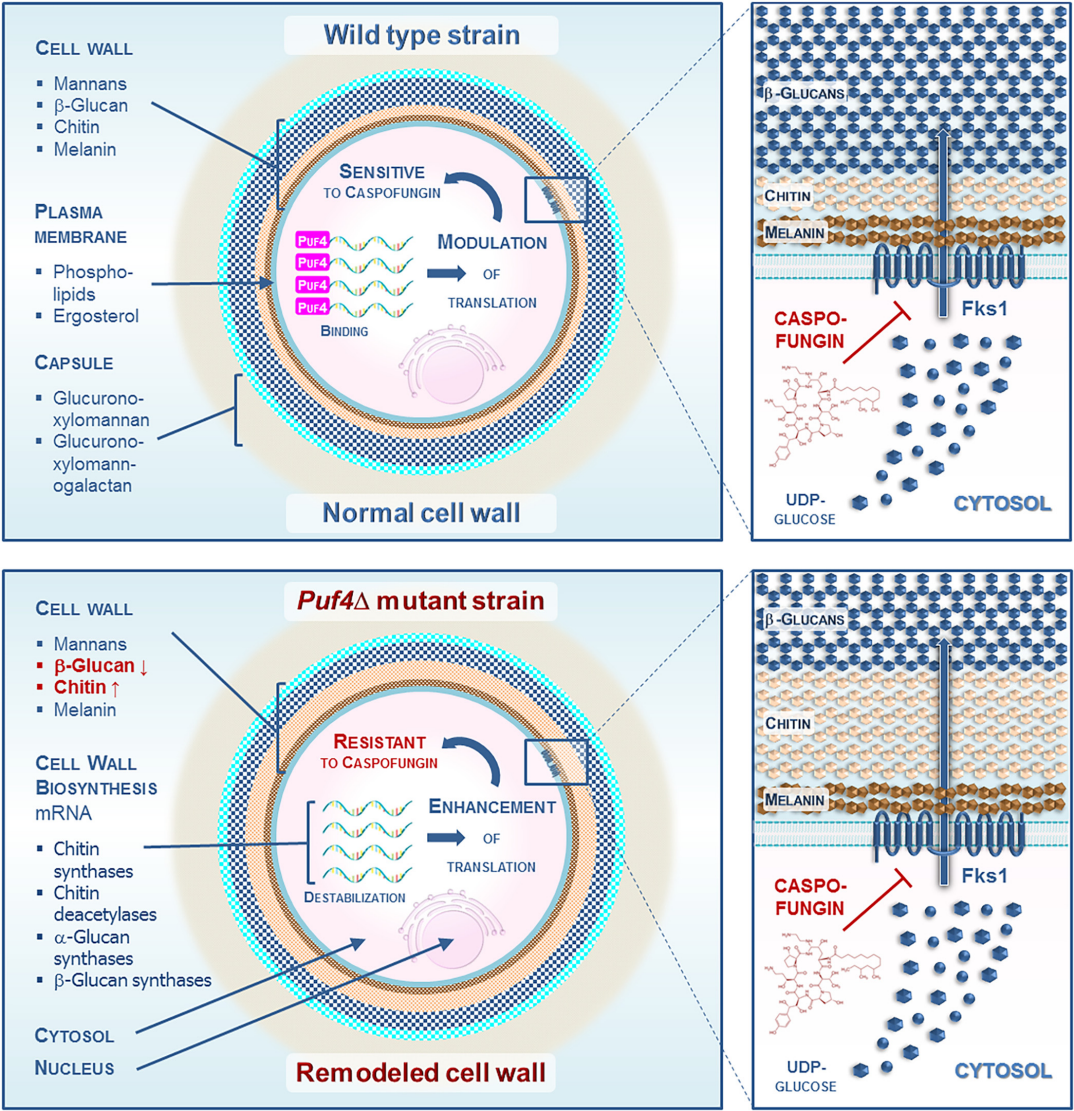
Cell wall biosynthesis genes and cell wall remodeling are posttranscriptionally regulated by Puf4 and govern caspofungin resistance in C. neoformans. In the wild-type strain, Puf4 binds cell wall biosynthesis mRNA and thus controls its stability and abundance. This participates in maintaining the cell wall composition and architecture. In the *puf4*Δ mutant strain, cell wall biosynthesis transcripts are destabilized. The lack of gene regulation by Puf4 induces a cell wall harboring more chitin and less β-1,3-glucan. All these posttranscriptional regulations of the cell wall biosynthesis transcripts influence caspofungin resistance in cells lacking Puf4.

Intriguingly, the investigators first observed that the *puf4*Δ mutant displayed a marked resistance to the echinocandin drug caspofungin compared to the wild-type strain. In addition, *PUF4* transcript and protein expression was shown to be decreased in the presence of caspofungin, suggesting the indirect influence of genetic interactions occurring between components of posttranscriptional gene regulatory networks on the drug resistance phenotypes. Then, the authors detailed the mechanism by which Puf4 directly binds and stabilizes the *FKS1* mRNA (encoding β-1,3-glucan synthase) by triggering a specific 5′ sequence called PBE (Puf4 binding element). Further experiments allowed Kalem and colleagues to nicely show that Puf4 may play a role in modulating not only *FKS1* expression but also the cell wall β-1,3-glucan contents. They provided molecular evidence that an increased translation of the *FKS1* mRNA is proportionally correlated with Fks1 protein abundance in a mutant lacking the *PUF4* gene. This means that Puf4 may act as a repressor of *FKS1* mRNA translation in C. neoformans. Interestingly, beyond binding *FKS1* mRNA, the Puf4 protein was also shown to directly interact with PBE, predicted to be within the sequence of a series of additional cell wall biosynthesis mRNAs, such as those encoding chitin synthases, chitin deacetylases, and α-glucan and β-glucan synthases. More specifically, the results shed light on multiple interactions that modulate the stability of cell wall biosynthesis mRNA, triggering either a positive or a negative effect on their translation during caspofungin treatment. In sum, all these results are consistent with a role of Puf4 as a master posttranscriptional regulator of cell wall remodeling that tunes caspofungin susceptibility. Finally, the structural relevance (i.e., cell wall composition changes) deriving from this complex Puf4-mediated molecular interplay was nicely revealed thanks to specific staining coupled with microscopy and flow cytometry approaches. In particular, this gave evidence that the absence of Puf4-mediated gene regulation creates a cell wall enriched in chitin and exposed chito-oligomers, while being devoid of β-1,3-glucan. Overall, this enlightening article provides an unprecedented view of how C. neoformans regulates caspofungin resistance through a complex posttranscriptional regulatory network (Fig. 1).

For more than 3 decades, the identification of fungal pathogenesis factors as new targets, the search for new biological and synthetic compounds with interesting antifungal activities, and deciphering molecular mechanisms involved in primary or acquired resistance toward antifungals have been part of a global strategy for antifungal drug development aiming ultimately at improving the management of deep-seated fungal infections (13, 14). Indeed, in the same way as antibiotic resistance in bacterial pathogens—but of course to a lesser extent—antifungal resistance has progressively become a serious clinical issue (15, 16). We know now that in medical mycology, this relies on two main features, namely, the extensive use of antifungal drugs for the prevention and treatment of an increasing number of immunocompromised patients (e.g., more patients with cancers or receiving transplants, etc.) and the emergence of naturally multiresistant fungal agents (e.g., Mucorales, Candida auris). A rapid overview of the literature teaches us that, globally, acquired resistances deriving from antifungal monotherapies usually involve a single to a couple of single nucleotide polymorphisms in the genomes of resistant isolates, suggesting that a few point mutations are sufficient to dynamically select resistant populations for surviving in a treated host organism. However, the context is sharply different in the case of primary resistance. While not deriving from drug exposure, underlying molecular mechanisms of primary resistance are mostly of multifactorial origins and were likely developed during the stepwise evolution and specialization of fungal lineages (17). As a consequence, unravelling the molecular determinants of intrinsic resistance in fungal pathogens is often laborious work. With such a perspective, we must recognize this excellent report by the research group of John C. Panepinto, additionally because of the contribution of posttranscriptional gene regulation in drug resistance phenotypes, which has hitherto remained largely unexplored in pathogenic fungi due to the substantial complexity of these cell processes. However, there is no need to argue that a better understanding of such complex regulatory networks may lead to the identification of key components that could be targeted by adjunctive therapies for improving the efficacies of currently available drugs (10, 18–20).

## References

[1] Fisher MC, Gurr SJ, Cuomo CA, Blehert DS, Jin H, Stukenbrock EH, Stajich JE, Kahmann R, Boone C, Denning DW, Gow NAR, Klein BS, Kronstad JW, Sheppard DC, Taylor JW, Wright GD, Heitman J, Casadevall A, Cowen LE. 2020. Threats posed by the fungal kingdom to humans, wildlife, and agriculture. mBio 11:e00449-20. doi:10.1128/mBio.00449-20. 32371596 PMC7403777

[2] Köhler JR, Hube B, Puccia R, Casadevall A, Perfect JR. 2017. Fungi that infect humans. Microbiol Spectr 5:eFUNK-0014-2016. doi:10.1128/microbiolspec.FUNK-0014-2016. PMC11687496 28597822

[3] Bongomin F, Gago S, Oladele RO, Denning DW. 2017. Global and multi-national prevalence of fungal diseases-estimate precision. J Fungi 3:57. doi:10.3390/jof3040057. PMC5753159 29371573

[4] Zhao Y, Lin J, Fan Y, Lin X. 2019. Life cycle of Cryptococcus neoformans. Annu Rev Microbiol 73:17–42. doi:10.1146/annurev-micro-020518-120210. 31082304 PMC12860491

[5] Casadevall A, Coelho C, Cordero RJB, Dragotakes Q, Jung E, Vij R, Wear MP. 2019. The capsule of Cryptococcus neoformans. Virulence 10:822–831. doi:10.1080/21505594.2018.1431087. 29436899 PMC6779390

[6] Kwon-Chung KJ, Fraser JA, Doering TL, Wang Z, Janbon G, Idnurm A, Bahn YS. 2014. Cryptococcus neoformans and Cryptococcus gattii, the etiologic agents of cryptococcosis. Cold Spring Harb Perspect Med 4:a019760. doi:10.1101/cshperspect.a019760. 24985132 PMC4066639

[7] Coelho C, Casadevall A. 2016. Cryptococcal therapies and drug targets: the old, the new and the promising. Cell Microbiol 18:792–799. doi:10.1111/cmi.12590. 26990050 PMC5536168

[8] Alspaugh JA. 2015. Virulence mechanisms and Cryptococcus neoformans pathogenesis. Fungal Genet Biol 78:55–58. doi:10.1016/j.fgb.2014.09.004. 25256589 PMC4370805

[9] Maligie MA, Selitrennikoff CP. 2005. Cryptococcus neoformans resistance to echinocandins: (1,3)beta-glucan synthase activity is sensitive to echinocandins. Antimicrob Agents Chemother 49:2851–2856. doi:10.1128/AAC.49.7.2851-2856.2005. 15980360 PMC1168702

[10] Bermas A, Geddes-McAlister J. 2020. Combatting the evolution of antifungal resistance in Cryptococcus neoformans. Mol Microbiol 114:721–734. doi:10.1111/mmi.14565. 32697029

[11] LeBlanc EV, Polvi EJ, Veri AO, Privé GG, Cowen LE. 2020. Structure-guided approaches to targeting stress responses in human fungal pathogens. J Biol Chem 295:14458–14472. doi:10.1074/jbc.REV120.013731. 32796038 PMC7573264

[12] Glazier VE, Kaur JN, Brown NT, Rivera AA, Panepinto JC. 2015. Puf4 regulates both splicing and decay of HXL1 mRNA encoding the unfolded protein response transcription factor in Cryptococcus neoformans. Eukaryot Cell 14:385–395. doi:10.1128/EC.00273-14. 25681267 PMC4385805

[13] Lee Y, Puumala E, Robbins N, Cowen LE. 22 5 2020. Antifungal drug resistance: molecular mechanisms in *Candida albicans* and beyond. Chem Rev doi:10.1021/acs.chemrev.0c00199. PMC8519031 32441527

[14] Xue A, Robbins N, Cowen LE. 28 8 2020. Advances in fungal chemical genomics for the discovery of new antifungal agents. Ann N Y Acad Sci doi:10.1111/nyas.14484. PMC8514151 32860238

[15] Revie NM, Iyer KR, Robbins N, Cowen LE. 2018. Antifungal drug resistance: evolution, mechanisms and impact. Curr Opin Microbiol 45:70–76. doi:10.1016/j.mib.2018.02.005. 29547801 PMC6135714

[16] Robbins N, Caplan T, Cowen LE. 2017. Molecular evolution of antifungal drug resistance. Annu Rev Microbiol 71:753–775. doi:10.1146/annurev-micro-030117-020345. 28886681

[17] Cowen LE. 2008. The evolution of fungal drug resistance: modulating the trajectory from genotype to phenotype. Nat Rev Microbiol 6:187–198. doi:10.1038/nrmicro1835. 18246082

[18] Ball B, Langille M, Geddes-McAlister J. 2020. Fun(gi)omics: advanced and diverse technologies to explore emerging fungal pathogens and define mechanisms of antifungal resistance. mBio 11:e01020-20. doi:10.1128/mBio.01020-20. 33024032 PMC7542357

[19] Papon N, Sanglard D. 2020. Tracking the origin and evolution of multidrug resistance in Candida auris. Lancet Microbe 1:e237. doi:10.1016/S2666-5247(20)30124-5. 35544217

[20] Kannan A, Asner SA, Trachsel E, Kelly S, Parker J, Sanglard D. 2019. Comparative genomics for the elucidation of multidrug resistance in Candida lusitaniae. mBio 10:e02512-19. doi:10.1128/mBio.02512-19. 31874914 PMC6935856

